# Quality of Life among Peritoneal and Hemodialysis Patients: A Cross-Sectional Study

**DOI:** 10.3390/clinpract13050109

**Published:** 2023-09-30

**Authors:** Fadel AlRowaie, Abdullah Alaryni, Abdullah AlGhamdi, Renad Alajlan, Razan Alabdullah, Raed Alnutaifi, Raneem Alnutaifi, Amani Aldakheelallah, Alanoud Alshabanat, Abdullah Bin Shulhub, Othillah Moazin, Rayan Qutob, Enad Alsolami, Osamah Hakami

**Affiliations:** 1 Nephrology Department, King Fahad Medical City, Riyadh 12231, Saudi Arabia; falrowaie@kfmc.med.sa; 2 College of Medicine, Imam Mohammad Ibn Saud Islamic University, Riyadh 11623, Saudi Arabia; aaalaryni@imamu.edu.sa (A.A.); ahoalghamdi@imamu.edu.sa (A.A.); dr.razanalabdullah@gmail.com (R.A.); raneemalnutaifi@gmail.com (R.A.); amani.aldakheelalla@gmail.com (A.A.); alshabanat.alanoud@gmail.com (A.A.); abdullahalshalhoub@gmail.com (A.B.S.); othillah09@gmail.com (O.M.); raqutob@imamu.edu.sa (R.Q.); oahakami@imamu.edu.sa (O.H.); 3 College of Medicine, King Saud University, Riyadh 11451, Saudi Arabia; raedalnutaifi10@gmail.com; 4 Department of Internal Medicine, College of Medicine, University of Jeddah, Jeddah P.O. Box 45311, Saudi Arabia; eaalsolami@uj.edu.sa

**Keywords:** peritoneal dialysis, hemodialysis, kidney disease quality of life, cross-sectional study, Saudi Arabia

## Abstract

Background: The quality of life (QoL) of patients with end-stage kidney disease (ESKD) who undergo dialysis is a reliable predictor of their long-term survival. Hemodialysis is the most common form of kidney replacement therapy for ESKD, followed by peritoneal dialysis. This study aimed to identify the factors affecting QoL in ESKD patients treated with peritoneal dialysis (PD) or hemodialysis (HD) in Riyadh, Saudi Arabia. Methods: A cross-sectional study was conducted between June and July 2021 to assess the QoL of patients with ESKD who underwent peritoneal dialysis and hemodialysis. Patients who had been on dialysis for at least one year were included. The Arabic version of the Quality of Life Index–Dialysis (QLI-D) version III was used to measure the QoL. Results: A total of 210 patients completed the questionnaire. The overall QLI score was 21.73 ± 4.2, with subscales for health and functioning (20.35 ± 5.2), social and economic (20.20 ± 4.8), psychological/spiritual (23.94 ± 4.9), and family (24.95 ± 4.5). The QLI scores for PD and HD patients were 21.80 ± 4.4 and 21.72 ± 4.1, respectively. SOCSUB (*p* = 0.031) was significantly associated with group and income, whereas QLI (*p* = 0.003), HFSUB (*p* = 0.013), SOCSUB (*p* = 0.002), and PSPSUB (*p* = 0.003) were significantly correlated with group and years of dialysis. Conclusion: The study found that patients were most satisfied with their family, health and functioning, and social/economic subscales. Income and years of dialysis were found to be predictive factors of QoL. Overall, peritoneal patients in this study demonstrated a better QoL than HD patients.

## 1. Introduction

The quality of life (QoL) of dialysis patients with end-stage kidney disease (ESKD) is an important factor affecting their overall health and well-being. The best dialysis modality for ESKD is still unresolved. Factors typically considered when making this decision include patient motivation and willingness, geographic location, doctor and caregiver bias, and patient education. Peritoneal dialysis (PD) is a treatment modality that offers patients greater autonomy and flexibility, allowing them to return to and maintain their daily activities better [1]. Psychological factors are also important in predicting patient compliance and QoL. Understanding how PD and hemodialysis (HD) affect patients’ lives is essential for the progression and management of ESKD.

It is essential to assess the health-related QoL of PD patients, as QoL is a crucial indicator of the overall well-being of a patient and can be used to monitor the efficacy of therapy and identify the areas where improvements can be made [2]. A 2017 meta-analysis identified seven studies that investigated QoL among patients undergoing HD and PD, with one of the studies reporting that patients with PD were more satisfied with their care, whereas those on HD were more satisfied with their physical condition post-therapy [3]. In 2019, 21,068 patients in Saudi Arabia received kidney replacement therapy (RRT), with 19,522 being on HD and 1546 on PD, according to the data published in the annual report of the Saudi Center for Organ Transplantation for the year 2020 [4]. A previous cross-sectional study conducted in Saudi Arabia found that PD-treated patients had a better overall QoL than those post-HD, except in the physical domain, where patients with HD scored higher [5]. However, PD-treated patients had a higher QoL than HD-treated patients concerning physical and psychological well-being [6]. 

The rates of depression among patients post-PD were significantly lower than before [7]. Similarly, a longitudinal study conducted in England found that male Asians with poor nutritional status were associated with lower QoL in PD-treated patients [8]. Social and economic status can also affect QoL. In addition, several studies have shown that comorbidities, hemoglobin levels, and dialysis quality (as measured by Kt/V) are also important factors. A prospective, randomized, double-blind, cross-over study was conducted to investigate the effects of epoetin on the QoL at different levels of hemoglobin (Hb); the results demonstrated that QoL significantly improved at Hb 14 compared to Hb 10 [9]. An observational study found that the baseline peritoneal Kt/V urea levels affected the QoL of patients after PD [10]. Another observational study investigated the factors associated with QoL. QoL was lower in patients with increasing age, cardiovascular disease, diabetes, and lower hemoglobin and Na levels [11].

This cross-sectional study aimed to identify the factors affecting QoL in ESKD patients treated with peritoneal dialysis (PD) or hemodialysis (HD) in Riyadh, Saudi Arabia. The level of satisfaction was assessed in four important life domains: health and functioning, social and economic status, psychological/spiritual beliefs, and family life

## 2. Materials and Methods

### 2.1. Study Design, Setting, and Participants

The study included adult patients aged 18 years or older with end-stage kidney disease (ESKD) who underwent PD and HD in Riyadh, Saudi Arabia, between June and July 2021. Patients younger than 18 years old or who did not consent to participate were excluded. PD-treated patients were recruited from the King Fahad Medical City (KFMC), whereas all the HD-treated patients were recruited from the King Salman Center for Kidney Disease (KSCKD).

### 2.2. Study Tool and Survey Administration

This study used the Arabic version of the Ferrans and Powers Quality of Life Index–Dialysis version III (QLI-D) to assess the QoL. The consent of the participants was obtained over the phone, and the questionnaire was prepared in an easy-to-understand format.

### 2.3. Quality of Life Index–Dialysis Version III

The QLI-D is a 33-item questionnaire that assesses the levels of satisfaction and importance concerning four domains: health and functioning, social and economic status, psychological/spiritual beliefs, and family life. The translated version of the QLI-D was found to have adequate content validity.

The satisfaction scale ranged from 1 (very dissatisfied) to 6 (very satisfied), and the importance scale ranged from 1 (very unimportant) to 6 (very important). The overall and subscale scores ranged from 0 to 30, with higher values indicating a better QoL.

### 2.4. Outcomes

The primary outcome of this study was to measure satisfaction and importance concerning the various domains of life.

### 2.5. Statistical Analysis

The data obtained were analyzed using SPSS version 23 (IBM Corp., Armonk, NY, USA) and visually represented using GraphPad Prism version 8 (GraphPad Software Inc., San Diego, CA, USA). Descriptive statistics were used to summarize the study variables, with categorical and nominal variables presented as counts and percentages and continuous variables presented as means and standard deviations. Pearson’s correlation coefficient was used to correlate the variables represented by means. Chi-square tests were used to establish the relationships between categorical variables. Independent *t*-tests were used to compare the mean values of the two groups. These tests were conducted assuming a normal distribution. General Linear Model (GLM) Multivariate Analysis was used to identify significant predictors using an interaction model. The null hypothesis was rejected at a *p*-value < 0.05.

## 3. Results

This cross-sectional study involved 210 participants who completed a questionnaire. The majority of respondents were between the ages of 51 and 70 years (42.9%), male (67.0%), married (65.7%), and had completed secondary school (37.8%). Of these, 37.2% were employed, 35.7% were unemployed, and 20.8% were retired; 43.2% earned less than 5000 Saudi riyals monthly. Regarding comorbidities, 53.8% had diabetes, 72.4% had hypertension, and 25.2% had cardiovascular disease. Additionally, 54.1% had been on dialysis for 1–5 years. Most participants (68.6%) received dialysis at the KSCKD, whereas the remaining 31.4% were at the KFMC. The participants were divided into two groups: those who received HD (82.4%) and those who received PD (17.6%). The results obtained are summarized in Table 1.

The overall mean QLI and subscale scores are summarized in Table 2. The QLI score was 21.73 ± 4.2, whereas the subscales for health and functioning, social and economic factors, psychological/spiritual status, and family life were 20.35 ± 5.2, 20.20 ± 4.8, 23.94 ± 4.9, and 24.95 ± 4.5, respectively. Table 2 indicates that the family subscale scored the highest of all. 

The relationship between QLI and subscales was determined at a statistical significance level of 0.01. QLI was significantly associated with HFSUB (health and functioning domain), SOCSUB (social and economic status domain), PSPSUB (psychological and spiritual belief domain), and FAMSUB (family life domain), (*p* < 0.001 of all). HFSUB had a significant relationship with SOCSUB, PSPSUB, and FAMSUB (*p* < 0.001 of all), whereas SOCSUB had a significant relationship with PSPSUB and FAMSUB (*p* < 0.001 of all). Furthermore, the findings revealed a significant relationship between PSPSUB and FAMSUB (*p* < 0.001), as shown in Table 3. 

Statistical analysis was also conducted on the QLI and subscale scores of the two PD and HD groups. The results revealed that the QLI of both the PD and HD groups were significantly associated with all subscales (*p* < 0.001 of all). HFSUB was significantly related to SOCSUB, PSPSUB, and FAMSUB (*p* < 0.001 of all), whereas SOCSUB was significantly related to PSPSUB and FAMSUB (*p* < 0.001 of all). Furthermore, the findings revealed a significant relationship between PSPSUB and FAMSUB (*p* < 0.001) in both the PD and HD groups (Table 4).

Table 5 illustrates the sociodemographic characteristics of the participants of the PD and HD groups. An analysis of the data showed that only income, years on dialysis, and dialysis center showed statistically significant differences between the two groups. The majority of the PD-treated patients (37.7%) earned 5000–10,000 Saudi Riyals, whereas most of the HD-treated patients (86.5%) earned < 5000 SR. In terms of years on dialysis, most PD-treated patients (37.0%) had been on dialysis for 0–1 year, whereas a majority of the HD-treated patients (80.4%) had been on dialysis for 1–5 years. The findings also revealed that all the patients in the PD-treated group came from the KFMC, whereas most of the HD-treated patients went to the KSCKD.

The mean QLI and subscale scores of the PD and HD groups are shown in Table 6 and illustrated in Table 2. The QLI scores for the PD and HD groups were 21.80 ± 4.4 and 21.72 ± 4.1, respectively. The HFSUB score of the PD group (19.35 ± 4.9) was lower than that of the HD patients (20.55 ± 5.3). Meanwhile, the SOCSUB score of the PD group was higher than that of the HD group (19.92 ± 4.6). Moreover, PD patients had higher PSPSUB (24.23 ± 4.9) and FAMSUB (25.74 ± 4.7) scores than HD patients (23.88 ± 4.9 and 24.78 ± 4.5). There were no significant differences between the two groups.

The results revealed that the statistically significant differences observed between the two groups were further analyzed using GLM Multivariate Analysis at a significance level < 0.05. Analysis of the data showed that only SOCSUB (*p* = 0.031) showed a significant association with group and income, whereas QLI (*p* = 0.003), HFSUB (*p* = 0.013), SOCSUB (*p* = 0.002) and PSPSUB (*p* = 0.003) showed a significant correlation with group and years of dialysis (Table 7). 

## 4. Discussion and Limitations 

The common treatment modalities for patients with ESKD are HD, PD, and kidney transplants, each of which has its benefits and drawbacks, as well as varying effects on the QoL of the patient. 

ESKD is associated with an increased risk of cardiovascular morbidity, mortality, and severe impairment in the QoL. There are three main treatment modalities for patients with ESKD: HD, PD, and kidney transplantation. Each modality has its benefits and drawbacks, and the effects on the QoL of the patients vary. The QoL of patients who underwent PD was investigated in the current study. The findings demonstrated relatively high QLI scores in four selected domains of life, indicating that respondents were relatively satisfied with them. Most participants were male and between the ages of 51 and 70. This was consistent with a previous study, which showed that men are more likely to develop kidney disease than women [5,8], which may be because of a faster decline in the function of kidneys in men than in women, which can harm their quality of life (HRQOL) [12]. 

Most of the participants were unemployed, which was not surprising, as many people with kidney disease find it difficult to work due to their treatment-related demands. Previous studies have shown that unemployment was high even among ESKD patients who had received successful kidney transplantation [13]. The findings of this study highlighted the need for more support for people with kidney disease, both in terms of medical care and employment. It is important to ensure that people with kidney disease have access to the necessary resources to live a full and productive life.

The QoL of patients with ESKD was assessed in this study using four subscales: health and functioning, social and economic stature, psychological/spiritual status, and family life. The results showed that overall QoL scores and the four subscales were relatively good. The mean score for the family subscale was the highest, whereas the mean score for the HFSUB and SOCSUB were the lowest, suggesting that participants were most satisfied with the family subscale and least satisfied with the HFSUB and SOCSUB ones.

The high satisfaction with the family subscale can be explained by family and friends providing much-needed help and support when a patient begins dialysis [14]. Kidney failure, which necessitates dialysis, requires changes in work schedules, to and from transportation, diet, and lifestyle. Family assistance is essential for dialysis patients to help them adapt to their new lifestyle. The low satisfaction with the health and functioning subscale is not surprising. Dialysis can significantly impact a patient’s life; however, it is time-consuming and often leaves patients exhausted post-completion. Although dialysis is a life-sustaining treatment, it requires a great deal of adaptation and adjustment on the part of the patient.

The low satisfaction with the social and economic subscale is supported by a previous study that reported poor satisfaction levels in the social and economic domains of ESKD patients receiving HD [15].

The participants of this study were divided into two groups: PD (*n* = 173) and HD (n = 37). The QLI scores for both groups were similar, but the participants of the PD group had higher scores in the social and economic, psychological/spiritual, and family subscale. This indicates that PD-treated patients were more satisfied with their overall quality of life than HD-treated patients.

The findings of this study are consistent with those of a previous research study, which has shown that PD-treated patients have a better quality of life in some domains than HD-treated patients [5,8,16,17]. This is likely due to the differences in the dialysis methodologies between the two treatment methods. HD-treated patients require visiting a treatment facility two to three times per week for four hours per session, which can harm their personal and professional lives. PD, on the other hand, can be performed at home or work, which gives patients more flexibility and freedom.

Previous research on the quality of life of PD- and HD-treated patients has been inconclusive. Some studies have found that PD has advantages in some domains, whereas others have found no differences between the two modes of dialysis [16,17]. The findings of the current study suggest that PD may have a better overall impact on QoL than HD, but more research is needed to confirm this.

This study investigated the factors associated with QoL in patients undergoing PD and HD. The study found that income and years spent on dialysis were predictive factors of QoL for PD and HD patients. Age affected the physical and social domains of QoL, whereas education impacted the environmental domain. Marital status was found to be related to the psychological and social domains [18]. Gender, age, ethnicity, social status, location and satisfaction post-dialysis, and causes of ESKD are all predictive factors of QoL [19].

The geographic location, accessibility to HD centers, acceptance of and adjustment to the situation, self-management, support from family members and care providers, and availability of properly trained nurses were all predictive factors of QoL [20]. In this study, income and years spent on dialysis were predictive factors of QoL for PD and HD patients.

This study, however, had some limitations. Firstly, it was a cross-sectional study, which means it was impossible to determine the direction of causality between the variables. Secondly, the study population was skewed toward HD patients, which may have affected the results. Furthermore, the study did not collect enough data on other factors that could affect the QoL, such as an extended list of possible comorbidities and social support and employment status. It is also worth mentioning that a single-center study limits generalization even with a relatively moderate sample size; multicenter and national studies will reflect a more accurate result. Although self-reported data are prone to bias, more clear and extensive objectives can limit such effects.

Despite these limitations, this study is significant because it is one of the few studies investigating the impact of income and years spent on dialysis on QoL in ESKD patients. These findings suggest that socioeconomic factors play an important role in QoL for patients with ESKD.

A longitudinal study would be useful to investigate further the relationship between income, years spent on dialysis, and QoL in patients with ESKD. Such a study would allow researchers to track patients over time and assess how their QoL changes in response to these factors.

## 5. Conclusions

This cross-sectional study investigated the QoL of PD and HD patients. The study found that patients had relatively high QoL scores, with the highest in the family subscale and the lowest in the health and functional and social/economic subscales. The study also found that income and years spent on dialysis were the predictive factors of QoL, with higher income and fewer years of dialysis being associated with a better QoL. Overall, in this study, the PD-treated patients had better QoL than the HD-treated patients.

This cross-sectional study investigated the QoL of PD and HD patients. This study pointed out that men are more likely to develop kidney disease than women. Moreover, the patients who had relatively high QoL scores had the highest scores in the family subscale and the lowest in the health, functional, and social/economic subscales. The study also found that income and years spent on dialysis were the predictive factors of QoL, with higher income and fewer years of dialysis being associated with a better QoL. The PD group had higher scores in social and psychological status and family life, indicating that these patients had better QoL than the HD-treated patients. It was also apparent the difficulty in maintaining a work life due to treatment-related demands, as most participants were unemployed. Dialysis can significantly impact a patient’s life, as it is time-consuming and often leaves patients exhausted post-completion. This study is remarkable since it is one of the few to examine how income and the number of years spent on dialysis affect ESKD patients’ quality of life. These results imply that socioeconomic considerations have a significant impact on the quality of life for ESKD patients.

## Figures and Tables

**Table 1:** Sociodemographic characteristics of 210 study samples.

Variables		Count	%
Total		210	100.0
Age	18–30	24	11.4
	31–50	79	37.6
	51–70	90	42.9
	>70	17	8.1
Gender	Male	140	67.0
	Female	69	33.0
	Missing	1	
Marital Status	Married	138	65.7
	Single	36	17.1
	Widowed	22	10.5
	Divorced	14	6.7
Education	Nothing	33	15.8
	Elementary school	17	8.1
	Middle school	23	11.0
	Secondary school	64	30.6
	University	62	29.7
	Postgrade	10	4.8
	Missing	1	
Employment	Employed	77	37.2
	Unemployed	74	35.7
	Retired	43	20.8
	Disabled	13	6.3
	Missing	3	
Income	<5000	89	43.2
	5000–10,000	53	25.7
	10,000–15,000	41	19.9
	>15,000	23	11.2
	Missing	4	
DM	Yes	113	53.8
	No	97	46.2
HTN	Yes	152	72.4
	No	58	27.6
CVD	Yes	53	25.2
	No	157	74.8
Years on dialysis	0–1 year	54	25.7
	1–5 years	82	39.0
	5–10 years	46	21.9
	>10 years	28	13.3
HD Center	KFMC	65	31.4
	KSCKD	142	68.6
	Missing	3	
Group	PD	37	17.6
	HD	173	82.4

**Table 2:** Overall mean QLI and subscale scores.

Variables	*N*	Min	Max	Mean	SD
QLI	209	7.18	29.21	21.73	4.1
HFSUBa	209	4.20	30.00	20.35	5.2
SOCSUBb	209	3.38	30.00	20.20	4.8
PSPSUBc	209	8.71	30.00	23.94	4.9
FAMSUBd	209	5.30	30.00	24.95	4.5

**Table 3:** Relationship of quality of life to four domains.

Correlations		HFSUBa	SOCSUBb	PSPSUBc	FAMSUBd
**QLI**	r	0.918 **	0.811 **	0.865 **	0.612 **
	*p*-value	<0.001	<0.001	<0.001	<0.001
	N	209	209	209	209
**HFSUBa**	r		0.608 **	0.707 **	0.433 **
	*p*-value		<0.001	<0.001	<0.001
	N		209	209	209
**SOCSUBb**	r			0.663 **	0.425 **
	*p*-value			<0.001	<0.001
	N			209	209
**PSPSUBc**	r				0.472 **
	*p*-value				<0.001
	N				209

** Correlation is significant at the 0.01 level (2-tailed).

**Table 4:** Relationship between QoL and subscales of PD and HD groups.

Group			HFSUBa	SOCSUBb	PSPSUBc	FAMSUBd
PD	QLI	r	0.916 **	0.893 **	0.852 **	0.824 **
		*p*-value	<0.001	<0.001	<0.001	<0.001
		N	36	36	36	36
	HFSUBa	r		0.729 **	0.639 **	0.691 **
		*p*-value		<0.001	<0.001	<0.001
		N		36	36	36
	SOCSUBb	r			0.745 **	0.639 **
		*p*-value			<0.001	<0.001
		N			36	36
	PSPSUBc	r				0.717 **
		*p*-value				<0.001
		N				36
HD	QLI	r	0.925 **	0.798 **	0.868 **	0.566 **
		*p*-value	<0.001	<0.001	<0.001	<0.001
		N	173	173	173	173
	HFSUBa	r		0.607 **	0.727 **	0.395 **
		*p*-value		<0.001	<0.001	<0.001
		N		173	173	173
	SOCSUBb	r			0.648 **	0.366 **
		*p*-value			<0.001	<0.001
		N			173	173
	PSPSUBc	r				0.419 **
		*p*-value				<0.001
		N				173

** Correlation is significant at the 0.01 level (2-tailed).

**Table 5:** Comparison of sociodemographic characteristics of PD and HD patients.

Demographics		Total	Group		*p*-Value
			PD	HD	
Total		210	37 (17.6%)	173 (82.4%)	-
Age	18–30	24	5 (20.8%)	19 (79.2%)	0.871
	31–50	79	13 (16.5%)	66 (83.5%)	
	51–70	90	15 (16.7%)	75 (83.3%)	
	>70	17	4 (23.5%)	13 (76.5%)	
Gender	Male	140	22 (15.7%)	118 (84.3%)	0.283
	Female	69	15 (21.7%)	54 (78.3%)	
Marital Status	Married	138	24 (17.4%)	114 (82.6%)	0.680
	Single	36	5 (13.9%)	31 (86.1%)	
	Widowed	22	4 (18.2%)	18 (81.8%)	
	Divorced	14	4 (28.6%)	10 (71.4%)	
Education	Nothing	33	6 (18.2%)	27 (81.8%)	0.222
	Elementary school	17	1 (5.9%)	16 (94.1%)	
	Middle school	23	3 (13.0%)	20 (87.0%)	
	Secondary school	64	9 (14.1%)	55 (85.9%)	
	University	62	14 (22.6%)	48 (77.4%)	
	Postgrad	10	4 (40.0%)	6 (60.0%)	
Employment	Employed	77	13 (16.9%)	64 (83.1%)	0.328
	Unemployed	74	15 (20.3%)	59 (79.7%)	
	Retired	43	9 (20.9%)	34 (79.1%)	
	Disabled	13	0 (0.0%)	13 (100.0%)	
Income	<5000	89	12 (13.5%)	77 (86.5%)	<0.001 ^a^
	5000–10,000	53	20 (37.7%)	33 (62.3%)	
	10,000–15,000	41	2 (4.9%)	39 (95.1%)	
	>15,000	23	3 (13.0%)	20 (87.0%)	
DM	Yes	113	19 (16.8%)	94 (83.2%)	0.741
	No	97	18 (18.6%)	79 (81.4%)	
HTN	Yes	152	28 (18.4%)	124 (81.6%)	0.621
	No	58	9 (15.5%)	49 (84.5%)	
CVD	Yes	53	10 (18.9%)	43 (81.1%)	0.783
	No	157	27 (17.2%)	130 (82.8%)	
Years on dialysis	0–1 year	54	20 (37.0%)	34 (63.0%)	<0.001 ^a^
	1–5 years	82	12 (14.6%)	70 (85.4%)	
	5–10 years	46	5 (10.9%)	41 (89.1%)	
	>10 years	28	0 (0.0%)	28 (100.0%)	
HD Center	KFMC	65	35 (53.8%)	30 (46.2%)	<0.001 ^a^
	KSCKD	142	0 (0.0%)	142 (100.0%)	

^a^—significant using Chi-Square Test at <0.05 level.

**Table 6:** Comparison of QoL and subscale scores of PD and HD patients.

Variables	Total	PD	HD	*p*-Value
QLI	209	21.80 ± 4.4	21.72 ± 4.1	0.971
HFSUBa	209	19.35 ± 4.9	20.55 ± 5.3	0.207
SOCSUBb	209	21.52 ± 5.4	19.92 ± 4.6	0.067
PSPSUBc	209	24.23 ± 4.9	23.88 ± 4.9	0.694
FAMSUBd	209	25.74 ± 4.7	24.78 ± 4.5	0.250

**Table 7:** Relationship between sociodemographic characteristics and QoL and subscales.

Source		Type III Sum of Squares	Df	Mean Square	F	*p*-Value
Corrected Model	QLI	376.635 ^a^	13	28.972	1.835	0.041 *
	HFSUBa	574.271 ^b^	13	44.175	1.719	0.060
	SOCSUBb	790.961 ^c^	13	60.843	3.193	<0.001 *
	PSPSUBc	560.720 ^d^	13	43.132	2.024	0.021 *
	FAMSUBd	334.724 ^e^	13	25.748	1.307	0.212
Intercept	QLI	27,837.059	1	27,837.059	1762.681	<0.001 *
	HFSUBa	23,936.512	1	23,936.512	931.377	<0.001 *
	SOCSUBb	25,773.458	1	25,773.458	1352.628	<0.001 *
	PSPSUBc	32,246.563	1	32,246.563	1513.450	<0.001 *
	FAMSUBd	37,805.290	1	37,805.290	1919.245	<0.001 *
Group * Income	QLI	52.351	6	8.725	0.552	0.768
	HFSUBa	130.180	6	21.697	0.844	0.537
	SOCSUBb	270.800	6	45.133	2.369	0.031 *
	PSPSUBc	74.113	6	12.352	0.580	0.746
	FAMSUBd	99.963	6	16.661	0.846	0.536
Group * Years on dialysis	QLI	298.360	5	59.672	3.779	0.003 *
	HFSUBa	383.067	5	76.613	2.981	0.013 *
	SOCSUBb	384.369	5	76.874	4.034	0.002 *
	PSPSUBc	393.559	5	78.712	3.694	0.003 *
	FAMSUBd	129.610	5	25.922	1.316	0.259
Group * HD Center	QLI	1.072	1	1.072	0.068	0.795
	HFSUBa	24.690	1	24.690	0.961	0.328
	SOCSUBb	18.480	1	18.480	0.970	0.326
	PSPSUBc	10.374	1	10.374	0.487	0.486
	FAMSUBd	22.151	1	22.151	1.125	0.290
Error	QLI	2968.982	188	15.792		
	HFSUBa	4831.627	188	25.700		
	SOCSUBb	3582.219	188	19.054		
	PSPSUBc	4005.653	188	21.307		
	FAMSUBd	3703.225	188	19.698		
Total	QLI	99,514.342	202			
	HFSUBa	89,768.809	202			
	SOCSUBb	87,443.914	202			
	PSPSUBc	121,323.296	202			
	FAMSUBd	130,703.290	202			
Corrected Total	QLI	3345.617	201			
	HFSUBa	5405.898	201			
	SOCSUBb	4373.181	201			
	PSPSUBc	4566.373	201			
	FAMSUBd	4037.949	201			

^a^. R Squared = 0.113 (Adjusted R Squared = 0.051) ^b^. R Squared = 0.106 (Adjusted R Squared = 0.044) ^c^. R Squared = 0.181 (Adjusted R Squared = 0.124) ^d^. R Squared = 0.123 (Adjusted R Squared = 0.062) ^e^. R Squared = 0.083 (Adjusted R Squared = 0.019) *—significant using General Linear Model Multivariate Test at <0.05 level.

## Data Availability

Data used in this study are available upon reasonable request from the corresponding author.
